# Relationship between perceived exertion and blood lactate concentrations during incremental running test in young females

**DOI:** 10.1186/2052-1847-7-5

**Published:** 2015-01-22

**Authors:** Daijiro Abe, Takayoshi Yoshida, Hatsumi Ueoka, Koji Sugiyama, Yoshiyuki Fukuoka

**Affiliations:** Biodynamics Laboratory, Center for Health and Sports Science, Kyushu Sangyo University, 2-3-1 Matsukadai, 813-8503 Higashi-ku, Fukuoka Japan; Department of Health and Sports Sciences, Graduate School of Medicine, Osaka University, 1-17 Machikaneyama, 560-0043 Toyonaka, Osaka Japan; Department of Environmental and Applied Physiology, Faculty of Environmental and Symbiotic Sciences, Prefectural University of Kumamoto, 3-1 Tsukide, 862-8502 Higashi-ku, Kumamoto Japan; Department of Health and Physical Education, Faculty of Education, Shizuoka University, 836 Ohya, 422-8529 Suruga-ku, Shizuoka Japan; Faculty of Health and Sports Science, Doshisha University, 1-3 Miyakodani, 610-0394 Kyotanabe, Japan

**Keywords:** Physical fitness, CR-10, OBLA, Training, RPE, LT

## Abstract

**Background:**

To investigate more practical handling of Borg’s ratings of perceived exertion (RPE) and category-ratio scale of RPE (CR-10), we evaluated interrelationships between RPE, CR-10, and blood lactate concentrations (bLa) during incremental treadmill running tests for young females with different aerobic fitness levels.

**Methods:**

Oxygen consumption, heart rate, bLa, RPE, and CR-10 were measured from distance runners (DR; n = 15), race walkers (RW; n = 6), and untrained females (UT; n = 11). These variables corresponding to the lactate threshold (LT) and onset of blood lactate accumulation (OBLA) were compared among these groups.

**Results:**

The UT had significantly lower RPE at LT than DR and RW, although the CR-10 at LT was not significantly different among these groups. The CR-10 at OBLA was significantly lower for the UT than DR. The relationship between bLa and CR-10 was approximated well by two linear regression lines in all groups. The bLa at the intersection only for the RW was significantly lower than that at LT, however, such intersections were observed at CR-10 = 3.1 to 3.2 without significant group differences. The CR-10 scores at LT and intersections were not significantly different in each group.

**Conclusion:**

These results suggested that an intersection between CR-10 and bLa was observed at the CR-10 score around three points of first half regardless of the aerobic fitness levels in young females, and such CR-10 scores would be associated with LT.

## Background

The blood lactate concentrations (bLa) have been used to monitor exercise intensity during both resistance and dynamic exercises [1, 2]. In particular, Seiler and Kjerland [3] showed that more than 75% of an entire training program was set at an exercise intensity corresponding to or under the individual lactate threshold (LT), even for elite rowers [4], junior national-level cross-country skiers [3], world-class cyclists [5], highly trained long distance runners [6, 7], and race walkers [7]. However, several invasive blood samplings are required to determine the exercise intensity corresponding to the individual LT or onset of blood lactate accumulation (OBLA). Thus, these indices are not always applied when coaching endurance athletes.

Indeed, bLa increase exponentially or polynomially during incremental exercise [8–11]. In contrast, heart rate (HR) or ratings of perceived exertion (RPE) increase linearly associated with an increase in exercise intensity [12–14]. These previous findings suggested that the HR, bLa, or RPE appeared to be practical for monitoring training intensities during training sessions. The RPE was originally proposed for ergonomic purposes to evaluate “overall” perceived exertion, physical stress, and exhaustion during physical effort [15, 16]. Borg et al. [17] further found a significant relationship between RPE and HR or bLa during arm and leg exercises.

A modified RPE, so-called category-ratio scale of RPE (CR-10), was proposed as a unique method to evaluate various clinical perceptions, including localized pain, fatigue, and perceived exertion during physical effort [18]. It is important to note that this unique index is closely correlated with the physiological variables, such as HR and bLa, during submaximal “dynamic” exercise [18]. However, both RPE and CR-10 are still subjective when monitoring the individual exercise intensities. Indeed, a considerable variability exists in the RPE scores corresponding to the ‘ventilatory threshold (VT)’ or LT, ranging from 10.2 to 16.5. [14, 19–23]. Such a variety of RPE scores should be derived from mixed effects of gender, age, training status, or muscle fatigue. This implies that comparing the data for RPE or CR-10 might be limited to homogeneous groups based on aerobic fitness levels [21, 23–27], training status [3, 13, 26–29], or healthy aged people [30, 31].

As described previously, bLa exhibits a gradual increase at light and moderate exercise intensities and a sharp increase at a heavy exercise intensity [8–11]. The CR-10 shows a linear increase in association with exercise intensity during dynamic exercise [12, 13]. Based on these observations, there exists a possibility of an intersection if the CR-10 is expressed as a function of bLa. Thus, we hypothesized that the relationship between CR-10 and bLa could be approximated by two regression lines using a non-linear least squares method, and further hypothesized that an intersection would appear around LT for young females with various aerobic fitness levels. Our third hypothesis was that a possible intersection between CR-10 and bLa would be explained by a particular CR-10 score in those participants, because perceptual effort expressed by CR-10 shows a linear increase in association with exercise intensity [12, 13]. To test these hypotheses, the purpose of this study was to evaluate the interrelationships between bLa, HR, RPE, and CR-10 among groups of young females with different aerobic fitness levels.

## Methods

### Subjects

This study included fifteen female distance runners (DR group), six female race walkers (RW group), and eleven untrained females as a control (UT group). It was quite difficult to recruit “pure” race walkers, because some of Japanese race walkers often train as “Ekiden” (long-distance road relay) runners. Thus, the sample size for the RW was smaller than other groups. Instead, the RW involved “pure” race walkers only. The UT was recruited from untrained female senior and graduate students, so that a significant age difference appeared (Table 1). The physical characteristics of these three groups are summarized in Table 1. All participants had no history of cardiovascular or metabolic disease. Each participant had a normal menstrual cycle as defined by regular periodicity and was not taking oral contraceptives. Testing during the follicular phase was completed on days 3-6 (day 1 = first day of menstrual flow). After providing detailed explanations of all procedures as well as possible risks and benefits of participation, all participants signed informed consent. This study conformed to the Declaration of Helsinki, and an ethical committee established in Prefectural University of Kumamoto approved all procedures in the present study.

**Table 1:** **Physical Characteristics and maximal oxygen uptake in all groups**

Groups	n	Age (year)	Height (cm)	Weight (kg)	***VO***_***2max***_(ml kg^-1^ min^-1^)
**UT**	11	23.3±2.9	158.0±3.4	51.1±5.2	35.2±2.3
**DR**	15	19.1^*^±1.0	160.3±4.5	50.1±6.3	56.5±4.4
**RW**	6	19.2^*^±1.1	158.4±5.7	50.3±2.8	49.1^*#^±3.4

Data are mean ± SD. UT; untrained control, DR; distance runners, RW; race walkers. ^*^ *vs.* UT (*p* < 0.01).^#^ *vs.* DR (*p* < 0.01).

### Exercise protocols and measurements

An incremental running test was administered using a motor-driven treadmill at 0% grade (Nishikawa Iron, Ltd., Japan). The initial treadmill velocity was set at 180 m · min^-1^ for 5-min for the DR and 80 m · min^-1^ for 5-min for the RW and UT. The treadmill velocity was then increased by 20 m · min^-1^ every 5-min until volitional exhaustion. It was obvious that the UT and RW walked on the treadmill at the initial stage, however, they were instructed to run at more than 100 m · min^-1^ for the purpose of this study. These different initial treadmill velocities and our instruction for running at more than 100 m · min^-1^ would not influence perceived exertion at LT, because Ekkekakis et al. [20] showed that a difference of the incremental protocol did not influence the perceived exertion at LT.

Oxygen consumption (*V*O_2_), carbon dioxide output (*V*CO_2_), and minute ventilation were analyzed using a computerized breath-by-breath measurement system (AE-310S, Minato Medical Science Co. Ltd., Osaka), which were calibrated before each measurement with room air and reference gas of known concentrations (O_2_ 15.22%, CO_2_ 5.17%, and N_2_ 79.61%). The mean *V*O_2_ during the final 1-min of each stage was regarded as the *V*O_2_ for that stage. The ratio of the *V*CO_2_ to *V*O_2_ was used to calculate the respiratory exchange ratio (RER; *V*CO_2_/*V*O_2_). During the incremental running test, the HR was continuously monitored by an electrocardiogram, and the mean HR value during the final 1-min of each stage was regarded as the HR for that stage. Scores for RPE and CR-10 were selected at the end of each stage from a scale (Table 2). When given criteria were met (e.g., a plateau or a drop in *V*O_2_, HR > 95% of age-predicted maximum [32], or RER > 1.1), the highest mean value of 1-min *V*O_2_ was regarded as the individual maximal oxygen uptake (*V*O_2max_) [33, 34]. In this matter, a plateau or a drop in *V*O_2_ was the primary criterion for determining the *V*O_2max_, but if not, HR over 95% of age-predicted maximum [32] or RER > 1.1 was used to interpret the attainment of the volitional exhaustion.

**Table 2:** **Category-ratio scale of perceived exertion (CR-10) and original RPE**

CR-10 scale	RPE scale
0 Nothing at all	6
0.5 Very, very weak (just noticeable)	7 Very, very light
1 Very weak	8
2 Weak (light)	9 Very light
3 Moderate	10
4 Somewhat strong	11 Fairly light
5 Strong (heavy)	12
6	13 Somewhat hard
7 Very Strong	14
8	15 Hard
9	16
10 Very, very strong (almost max)	17 Very hard
^*^Maximal	18
	19 Very, very hard
	20

After selecting RPE and CR-10 scores, the participants put their right hand on a small table located beside the treadmill, and 20 μL blood samples were obtained from the right index finger. After blood sampling, bLa was analyzed by a lactate analyzer (Diagluca, HEK-30 L, Toyobo, Japan) using an enzyme electrode method. The individual LT was systematically determined on a basis of the log-log transformation for the relationship between bLa and *V*O_2_[7, 9, 35, 36]. This procedure was used to determine the value of each independent variable corresponding to the individual LT. *V*O_2_, %*V*O_2max_, HR, RER, RPE, bLa, and CR-10 corresponding to the individual LT were evaluated for each participant.

The onset of blood lactate accumulation (OBLA), defined as the exercise intensity corresponding to 4 mM bLa, was also determined on a basis of a method described by Abe et al. [8]. For instance, all variables obtained from the incremental test were plotted as a function of bLa. An exponential interpolation was applied for the relationship between bLa and each variable to determine the value of each variable corresponding to OBLA.

### Statistical analysis

Data are given as means ± standard deviations (SD). As explained before, a sample size of the RW was smaller than other groups, so that the standard error of the mean or measurement (SEM), which represents a within-participant deviation [37, 38], was also presented for the CR-10 and RPE scores corresponding to the possible CR-10 intersection and/or LT to estimate a range for the “true” mean value. The SD divided by √*n* gives the SEM, where *n* is the number of participants for each group. This procedure was done in the limitation section to provide absolute reliability [38]. A regression analysis using an exponential function was used to determine the relationships between bLa and RPE. Non-linear least squares analysis was also used to determine the relationship between CR-10 and bLa. This procedure gives an intersection between CR-10 and bLa, and the individual CR-10 corresponding to the intersection were compared to those obtained at LT. A one-way repeated measures analysis of variance (ANOVA) within participants was used to compare physical and physiological indices among three groups. A two-way repeated measures of ANOVA within participants (3 *groups* × 2 *CR-10 scores* at LT and intersections) was applied to compare the CR-10 at LT and at the intersections between CR-10 and bLa. The values for bLa obtained at LT and at the intersections were also compared using two-way repeated measures of ANOVA within participants. If a significant *F* value was obtained, then *Tukey’s* multiple comparison was used as a post hoc test for the appropriate data sets. Statistical significance was set at the 0.05 probability level.

## Results

The mean *V*O_2max_ for each group are summarized in Table 1. A significant difference was found in the *V*O_2max_ among three groups (DR > RW > UT, *F* =13.48, *p* < 0.0001). These significant differences in the *V*O_2max_ reflected differences in the %*V*O_2max_ at LT and OBLA (F = 5.00, *p* = 0.007 at LT and *F* = 3.33, *p* = 0.027 at OBLA, Table 3). Physical characteristics, such as body height and weight, were not significantly different among these groups (Table 1), however, the UT was significantly older than RW and DR (*p* = 0.007 and *p* < 0.0001 for the DR and RW, respectively).

**Table 3:** **Physiological and perceptual data obtained data obtained at lactate threshold (LT) and onset of blood lactate accumulation (OBLA)**

	UT	DR	RW
Variables			
**at LT**			
*VO*_*2*_ (ml kg^-1^ min^-1^)	19.5±3.1	43.6±4.0^**^	34.8±3.3^**#^
*%VO*_2max_ (%)	55.3±6.0	77.0±3.6^**^	70.8±2.3^**#^
HR (beats min^-1^)	137±14.1	162±12.3^**^	159±9.7^**^
RER	0.95±0.04	0.91±0.02^**^	0.89±0.02^**^
bLa (mM)	1.7±0.6	1.4±0.4	1.5±0.3
RPE	11.2±1.5	12.3±1.6^*^	13.0±1.6^*^
CR-10	3.18±0.87	3.40±0.83	3.83±1.17
**at OBLA**			
*VO*_2_ (ml kg^-1^ min^-1^)	30.6±3.2	53.0±4.0^**^	47.5±3.8^**^
%*VO*_2max_ (%)	87.4±9.4	93.7±3.0^*^	96.7±4.1^*^
HR (beats min^-1^)	180±9.4	180±3.0	185±7.4
RER	1.04±0.02	0.99±0.02^*^	0.98±0.01^*^
RPE	15.6±2.1	16.7±1.8	16.9±1.8
CR-10	5.8±1.5	7.0±1.3^*^	6.8±1.6

Data are mean ± SD. UT; untrained control, DR; distance runners, RW; race walkers, *VO*_2_; oxygen uptake, HR; heart rate, RER; respiratory exchange ratio, bLa; blood lactate concentration, CR-10; category- ratio scale of RPE, and RPE; ratings of perceived exertion. ^*^ and ^**^ *vs.* UT (*p* < 0.05 and p < 0.01), and ^#^ *vs.* DR (*p* < 0.01), respectively.

**Figure 1:**
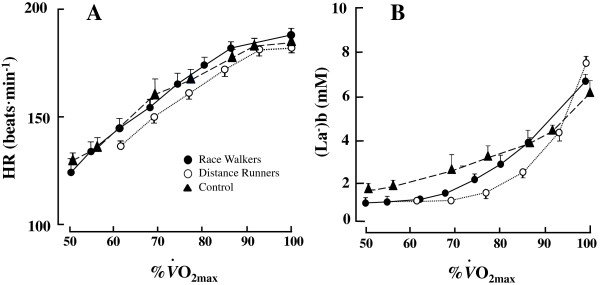
**Heart rate (HR, panel-A) and blood lactate concentrations (bLa, panel-B) expressed as a function of %** ***V*** **O**_**2max**_ **.** Distance runners (○), race walkers (●), and untrained individuals (▴). Values are means ± SD.

The HR responses during incremental test are shown in Figure 1A. The HR increased linearly as a function of %*V*O_2max_ (Figure 1A). Table 3 showed that the mean HR at LT was significantly lower for the UT than that for the DR and RW (*p* < 0.0001, Table 3), whereas the mean HR at OBLA was not significantly different among these groups (*p* = 0.13, Table 3).

**Figure 2:**
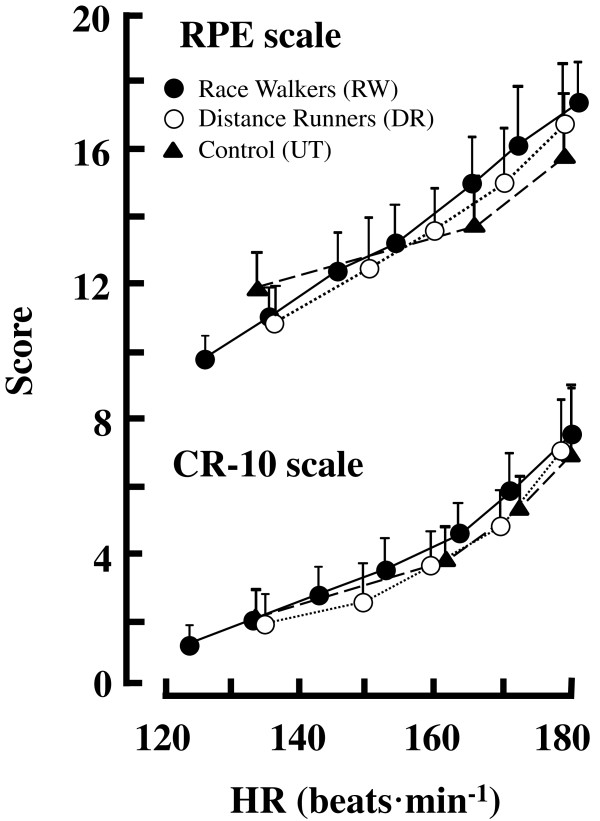
**RPE (upper) and CR-10 (lower) expressed as a function of HR. Each plot was same as Figure** 1 **.** Values are means ± SD.

**Figure 3:**
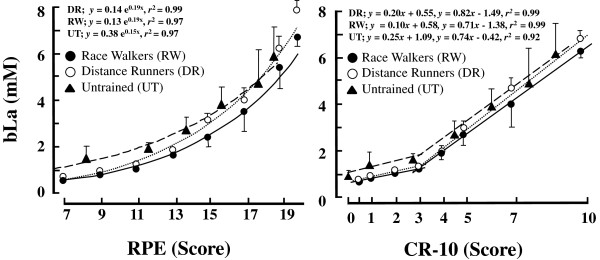
**RPE (left) and CR-10 (right) expressed as a function of bLa among three groups.** The curvilinear model between bLa and RPE and double regression lines between bLa and CR-10 could be applied. Each plot was same as Figure 1. Values are means ± SD.

During the incremental test, the RPE and CR-10 scores increased linearly as a function of HR for all groups (Figure 2). As shown in Table 3, the UT showed a significantly lower RPE at LT than the DR (*p* = 0.022) and RW (*p* = 0.013), whereas there were no significant differences in the CR-10 at LT and at the intersections (*p* = 0.12, Table 3 and Figure 3). In contrast, a significantly higher CR-10 at OBLA was found for the DR than for the UT (*p* = 0.007, Table 3), although there were no significant differences in the RPE at OBLA among these groups (*F* = 1.93, *p* = 0.07, Table 3).

**Figure 4:**
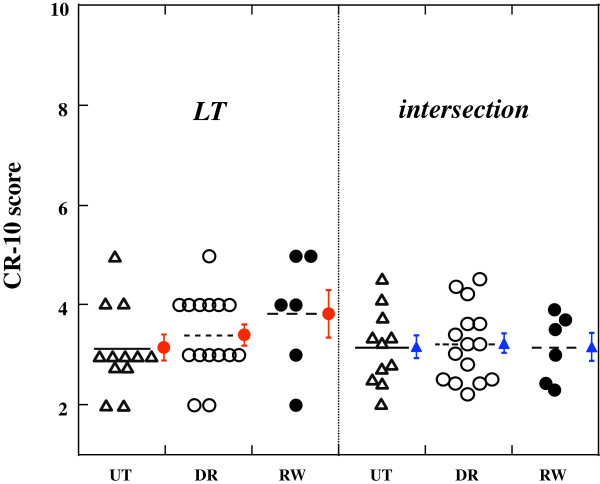
**A scattered graph of CR-10 scores obtained at LT and intersections between CR-10 and bLa.** Each plot is same as Figure 1. Solid, dashed, and dotted cross bars are the mean values of each group. Red and blue vertical bars are the SEM (within-participant variation) of each group.

The bLa expressed as a function of %*V*O_2max_ exhibited a curvilinear relationship during the incremental test for all groups (Figure 1B). The left panel of Figure 4 shows the curvilinear relationships between RPE and bLa for all groups. The relationships between CR-10 and bLa exhibited two regression lines with an intersection during the incremental test for all groups (Figure 4 right panel). Its determining variables (*r*^2^ values for two regression lines) constituted a coefficient more than 92%.

Figure 3 shows scattered CR-10 scores at LT and intersections. There were no significant differences in the CR-10 scores at the intersection among three groups (DR = 3.21 ± 0.75, RW = 3.13 ± 0.68, and UT = 3.14 ± 0.76, respectively, Figure 3 right side). A two-way ANOVA for 3 *groups* × 2 *CR-10 scores* revealed that no significant differences were found for the CR-10 scores at LT and intersections (score effect *p* = 0.97, group effect *p* = 0.87, groups × score interaction *p* = 0.75, Figure 3). Instead of the CR-10, the bLa at the intersections were significantly different among three groups (DR = 1.2 ± 0.4 mM, RW = 0.9 ± 0.3 mM, and UT = 1.9 ± 0.3 mM, respectively. *F* = 7.98, *p* < 0.0001). The bLa only for the RW at the intersection was significantly lower than that obtained at LT (*p* = 0.025).

## Discussion

### Overview

We investigated the interrelationships among HR, RPE, CR-10, and bLa for young females with different aerobic fitness levels. Major findings of this study were summarized as follows. 1. For all groups, the relationship between bLa and CR-10 was approximated well by two linear regression lines, indicating that an intersection was obtained for this relationship (Figure 4 right panel).

2. The mean bLa values at the CR-10 intersections, ranging from 0.9 to 1.9 mM, were significantly different among groups (Figure 4 right panel), and the bLa at the intersection for the RW was significantly lower than that obtained at LT (Table 3).3. Any significant differences were not observed in the CR-10 scores at LT and intersections (Figure 3).
4. The HR and RPE at OBLA were not significantly different among three groups, whereas the HR and RPE at LT were significantly lower for the UT than both endurance groups (Table 3).

### Interrelationship among physiological and perceptual variables at LT

In support of our first hypothesis, a considerable finding of the present study was that the relationship between bLa and CR-10 was approximated well by two linear regression lines, indicating that an intersection could be identified for this relationship for each group (Figure 4 right panel). It was interesting to note that the mean CR-10 at these intersections ranged from 3.1 to 3.2 without any group differences (right panels of Figures 4 and 3). However, the bLa at the intersection was significantly lower than that at LT only for the RW group, so that our second hypothesis was partly rejected. With respect to the CR-10 at the intersection, our third hypothesis was supported.

Table 3 showed that there were no statistically significant differences in the mean bLa at LT, which were equivalent to those obtained in a series of our previous studies [7, 35, 36]. The bLa at the intersection corresponded to 0.9-1.9 mM with significant group differences, and the bLa for the RW was significantly lower than that at LT. Thus, our first hypothesis with regard to the bLa values at the intersections was not supported. However, it was worth noting that the CR-10 at LT and intersections were not significantly different among three groups (Figure 3), suggesting that “CR-10 ≈ 3” might be a non-invasive criterion for evaluating the individual LT for young females regardless of their aerobic fitness levels. Indeed, “CR-10 ≈ 3” was further supported by previous studies in healthy older people [30, 31] and junior national-level cross-country skiers [3], although the ages and aerobic fitness levels of the participants widely ranged in those studies. A subjective variable (CR-10) in association with a physiological variable (bLa) would make us more confident for predicting exercise intensity corresponding to the individual LT, because it was independent of the aerobic fitness levels among young females (Figure 4, Figure 3, and Table 3).

Figure 2 showed that RPE (upper) and CR-10 (lower) in association with HR increased linearly during the incremental test for all groups. These linear responses for RPE and CR-10 during the incremental test were supported by the results of previous studies [12, 13, 17]. The mean CR-10 at LT ranged from 3.2 to 3.8 without any significant differences among these groups (Figure 3 and Table 3). Thus, these results indicated that the CR-10 could be useful to monitor the exercise intensity corresponding to the individual LT regardless of the aerobic fitness levels in young females. The perceptual efforts expressed by CR-10 at LT corresponded to “somewhat strong” in this study (Table 2). The CR-10 at LT seemed to be somewhat lower than that of some previous studies [24–27], but it was equivalent to that observed in other studies [3, 13, 30, 31]. These inconsistent results with regard to CR-10 at LT could be due to differences in participants’ disease state, such as COPD [24] and peripheral arterial disease [25] or the type of exercise [26, 27].

We also found that RPE at LT were significantly lower for the UT than for two endurance groups (Table 3). The perceptual efforts expressed by RPE at LT ranged from “fairly light” to “somewhat hard” (Table 2). However, no significant differences were found in the RPE and HR at LT between RW and DR, so that the data for RPE and HR could be also useful to predict the exercising intensity corresponding to the individual LT for female endurance athletes.

### CR-10 and RPE at OBLA

There is little information for CR-10 corresponding to the individual OBLA during dynamic exercise, particularly for females. As shown in Figure 1A, the HR increased linearly at submaximal exercise intensities for all groups. Table 3 showed that the HR at OBLA corresponded to around 180 beats · min^-1^ without any significant differences among three groups. There were no significant differences in the RPE at OBLA among three groups. These results indicated that a combination of HR and RPE could be useful to predict the exercise intensity corresponding to the individual OBLA regardless of the aerobic fitness levels. In fact, Borg et al. [17] suggested that a combination of HR and bLa could predict RPE more accurately than either variable alone when these variables were applied to moderately or highly fit males.

As explained in the methodological section, the RW and UT started walking on the treadmill at the initial velocity (80 m · min^-1^) for 5-min, and they were instructed to run at more than 100 m · min^-1^. Therefore, it was obvious that the individual walk-to-run transition (WRT) speed could be involved in a series of the incremental treadmill test. Indeed, the WRT influenced overall RPE [39] or CR-10 [40]. In our present study, the mean HR values at LT were 137 ± 14.1 beats · min^-1^ and 159 ± 9.7 beats · min^-1^ for the UT and RW, respectively (Table 3). These HR values corresponded to the CR-10 scores around 2 for the UT and 4 for the RW (Figure 2 lower panel), suggesting that the WRT does not influence the CR-10 or bLa at LT.

The CR-10 at OBLA was significantly higher for the DR than that for the UT, but it was not significantly different from that for the RW (Table 3). Thus, this combination could be particularly valuable for female endurance athletes when using CR-10 at OBLA. Fabre et al. [32] used a different method to determine individual exercise intensity at OBLA for highly fit professional soccer players. It is important to note that two previous studies [17, 32] had something in common as both involved trained athletes. Recent clinical investigations also revealed that a high-intensity interval training, which should be above OBLA, improved not only cardiorespiratory functions [41] but also locomotor functions [42] even in clinical patients. Thus, the determination of the individual exercise intensity at OBLA could be necessary not only for trained athletes but also for untrained or clinical patients.

### Practical applications

As previously described, endurance training models at LT or OBLA have been used in a number of studies and demonstrated significant improvements in the aerobic fitness level or performances not only for healthy older sedentary populations [30, 31] but also for different groups of endurance athletes [3, 7, 29, 43]. These previous studies indicated that programming LT training would be particularly important, and monitoring the training intensity corresponding to the individual LT would be necessary to lead potential athletes toward success in each endurance event.

The results of the present study will contribute to a practical handling of RPE and CR-10 for daily training not only for female endurance athletes but also for untrained females regardless of their aerobic fitness levels. However, some considerations are still necessary. Patients with cardiorespiratory disorders showed relatively greater RPE at LT than healthy populations [14, 24]. The RPE corresponding to the LT or VT seems to be different between males and females regardless of their fitness levels [14, 19, 21]. Unfortunately, our present study did not involve male participants, however, we showed a narrow range of CR-10 scores (3.1-3.2) at the intersection with a small deviation (coefficient of variance < 10%), being independent of their aerobic fitness levels in young females. The use of perceived exertion in association with HR and/or bLa will be useful for avoiding a risk of overtraining and a lack of necessary training intensity, at least, for females.

### Limitations

Since this study is a methodological study for the CR-10 intersection and its relevance to the LT, a reliability of the measured values and sample size should be further considered as a limitation of this study.

It is well known that the RPE is correlated to the heart rate [15]. A significantly lower RPE score was observed at LT only for the UT (Table 3 upper panel), but such a significantly lower RPE score for the UT should be associated with their HR at LT (Table 3 upper panel). Thus, a significant difference in the mean RPE score at LT between DR and UT (mean difference = 1.1) could be physiologically meaningful. In contrast, a non-significant difference of the RPE score at LT between DR and RW (mean difference = 0.7) should be carefully considered, because the mean HR at LT was equivalent in those endurance groups (Table 3). A 0.7 difference in the RPE score rounded off to one decimal place is 1.0. This is a practical problem. Hopkins [37] and Atkinson and Nevill [38] explained that the standard error of the mean or measurement (SEM) has been known as a within-participant deviation, which is substantially related to the “true” mean value. If the SEM is considered for such a non-significant difference (0.7), the “true” mean RPE should be at most 12.7 for the DR and at least 12.4 for the RW. These RPE scores ranged around 12-13 at LT for female endurance athletes are supported by some previous investigations [19, 20, 22].

The CR-10 scores at the intersections (mean ± SD) were 3.21 ± 0.75 for the DR, 3.13 ± 0.68 for the RW, and 3.14 ± 0.76 for the UT, respectively. When the SEM is considered, the “true” mean CR-10 scores at the intersections are expected to range 3.01-3.40 (DR), 2.85-3.41 (RW), and 2.91-3.37 (UT) (Figure 3 right side). Thus, the “CR-10 ≈ 3” for the intersection is justified for each group. In contrast, the CR-10 scores at LT (mean ± SD) are 3.40 ± 0.83 for the DR, 3.83 ± 1.17 for the RW, and 3.18 ± 0.87 for the UT. Thus, the “true” mean CR-10 scores at LT are expected to range 3.19-3.61 (DR), 3.35-4.31 (RW), and 2.92-3.44 (UT) (Figure 3 left side) if the SEM is considered. Although these results were almost equivalent to those obtained in a group of cross-country skiers [3], there is a possibility that “CR-10 ≈ 3” for LT may not be justified in female endurance athletes. Indeed, bLa at the intersection was significantly lower than that at LT only for the RW. It was interesting to note that such a significant difference was observed only for the RW whose number of participants was the smallest among three groups. This argument justifies the number of participants and statistical power used in the present study.

## Conclusions

The relationship between bLa and CR-10 was approximated well by two linear regression lines in all groups. The CR-10 scores at the intersections were within a narrow range of 3.1-3.2. The CR-10 scores at LT and intersections were not significantly different among three groups. Although the bLa at the intersection only for the RW was significantly lower than that at LT, the CR-10 scores at LT and intersections were not significantly different in each group. These results suggested that an intersection between CR-10 and bLa was observed at the CR-10 score around three points of first half regardless of the aerobic fitness levels in young females, and such CR-10 scores would be associated with LT.

The HR at OBLA was not significantly different among three groups, whereas CR-10 at OBLA was significantly lower for the UT than for the endurance athletes. These results further suggested that the CR-10 in association with HR responses could be also available to predict the exercising intensity corresponding to the individual OBLA for female endurance athlete.
